# Arecoline suppresses epithelial cell viability by upregulating tropomyosin-1 through the transforming growth factor-β/Smad pathway

**DOI:** 10.1080/13880209.2020.1851729

**Published:** 2020-12-17

**Authors:** Long Li, Liqun Gu, Zhigang Yao, Yuehong Wang, Zhangui Tang, Xiaoying Wu

**Affiliations:** a Department of Pathology, Xiangya Hospital, Central South University, Changsha, China; b Department of Pathology, School of Basic Medical Science, Central South University, Changsha, China; c Xiangya Stomatological Hospital, Central South University, Changsha, China; d Xiangya School of Stomatology, Central South University, Changsha, China

**Keywords:** Oral submucous fibrosis, HaCaT, cell-cycle arrest, apoptosis

## Abstract

**Context:**

Oral submucous fibrosis (OSF) is a chronic and progressive disease. Arecoline, present in betel nuts, has been proposed as a vital aetiological factor. However, the underlying mechanism remains unclear.

**Objectives:**

This research elucidates the expression of tropomyosin-1 (TPM1) and its regulation mechanism in HaCaT cells treated with arecoline.

**Materials and methods:**

HaCaT cells were assigned into three groups: (1) Control; (2) Treated with arecoline (0.16 mM) for 48 h (3) Treated with arecoline (0.16 mM) and transfected with small interfering RNA (siRNA) for TPM1 (50 nM) for 48 h. CCK8, cell cycle, and apoptosis phenotypic analyses were performed. PCR and western blot analyses were performed to detect the expression level of TPM1 and examine the related signalling pathway.

**Results:**

The IC_50_ of arecoline was approximately 50 μg/mL (0.21 mM). The arecoline dose (0.16 mM) and time (48 h) markedly increased TPM1 expression at the mRNA and protein levels in HaCaT cells. Arecoline suppressed the cell growth, caused cell cycle arrest at the G1 phase, and induced cell apoptosis in HaCaT cells. siRNA-mediated knockdown of TPM1 attenuated the effect of arecoline on cell proliferation, apoptosis, and cell cycle arrest at the G1 phase. Furthermore, blocking of the transforming growth factor (TGF)-β receptor using SB431542 significantly suppressed TPM1 expression in the cells treated with arecoline.

**Discussion and conclusions:**

Arecoline suppresses HaCaT cell viability by upregulating TPM1 through the TGF-β/Smad signalling pathway. This research provides a scientific basis for further study of arecoline and TPM1 in OSF and can be generalised to broader pharmacological studies. TPM1 may be a promising molecular target for treating OSF.

## Introduction

Oral submucous fibrosis (OSF) is a chronic precancerous disease characterised by the accumulation of collagen fibres in the submucosa and by epithelium atrophy. OSF causes pallor, burning sensation of the oral mucosa, and difficulty in speaking and swallowing (Le et al. 1996). The incidence of cancer in OSF ranges from 7% to 13% (Murti et al. 1985; Arakeri et al. 2017). Although epithelial atrophy and atypical epithelia can exist or coexist in OSF, their respective effects on malignant potential require further investigation.

The aetiology of OSF is exceedingly complex and aetiological factors have been proposed, including areca nut chewing, nutritional deficiencies, immunologic and genetic processes, and ingestion of chillies (Ahmad et al. 2006; Mohammed et al. 2015). Arecoline extracted from the areca nut is proposed as the paramount cause of OSF (Sumeth Perera et al. 2007; Hosthor et al. 2014). Arecoline affects cell viability in various ways, including interference with cell cycle progression (Thangjam and Kondaiah 2009; Chang et al. 2016; Gu et al. 2019), promotion of cell apoptosis (Lin et al. 2016; Peng et al. 2017), and inhibition of DNA damage repair (Tsai et al. 2008; Huang et al. 2016). However, the mechanisms of arecoline in epithelium atrophy and atypical hyperplasia remain unclear.

Tropomyosins (TMs) are a family of actin-binding proteins. There are four distinct genes: TPM1, TPM2, TPM3, and TPM4 (Lin et al. 2008; Schevzov et al. 2011). The TM protein family plays an essential role in altering stress fibres. Previous studies have demonstrated that TM-mediated actin fibres actively participate in extracellular matrix (ECM) deposition and remodelling and promote tissue fibrosis (Pawlak and Helfman 2001; Safina et al. 2009). TM is considered a marker of activated stellate cells in liver fibrosis (Otogawa et al. 2009). In addition, TPM1/2 is associated with remodelling of the actin cytoskeleton during epithelial-to-mesenchymal of lens epithelial cells and it was demonstrated that selective elevation of TPM1/2 is correlated with fibrosis observed in posterior capsule opacification (Kubo et al. 2013). In interstitial fibrosis, downregulation of the antifibrotic microRNA miR-29c was associated with significantly increased TPM1 expression (Fang et al. 2013). We aimed to determine the expression of TPM1 and its regulation mechanism in OSF.

## Materials and methods

### Cell culture

Oral keratinocytes have been frequently replaced by the HaCaT cell line in previous studies of oral disease (Khan et al. 2015; Peng et al. 2017). Therefore, we chose this cell model in the present study.

The HaCaT cell line was purchased from the National Infrastructure of Cell Line Resource (Beijing, China). The cells were cultured in Dulbecco’s modified Eagle’s medium (DMEM) supplemented with 10% foetal bovine serum (FBS; Hyclone, Auckland, NZ) in an environment of 5% CO_2_.

Arecoline hydrobromide (methyl 1-methyl-1,2,5,6-tetrahydronicotinate hydrobromide) was obtained from Sigma-Aldrich (St. Louis, MO, USA). SB431542 (a selective inhibitor of the transforming growth factor-beta 1 [TGF-β1] receptor) was obtained from Selleck Chemicals (Houston, TX, USA).

### Cell transfection

Cells were plated at a density of 5 × 10^5^ cells/well for 24 h prior to transfection. Once cells reached 75% confluence, they were transfected with 50 nM of synthesised small interfering RNAs (siRNAs) or scrambled controls using ribo*FECT*™ CP (Ribobio, Guangzhou, China) in each well of 6-well plates. The transfections were performed for 48 h. The siRNA sequences were as follows: si-h-TPM1_001: forward, 5′-GACGTAGCTTCTCTGAACA dTdT-3′; reverse, 3′-dTdT CTGCATCGAAGAGACTTGT-5′; si-h-TPM1_002: forward, 5′- GCGGAGAGGTCAGTAACTA dTdT-3′; reverse, 3′-dTdT CGCCTCTCCAGTCATTGAT-5′; and si-h-TPM1_003: forward, 5′-GTAAGCTGGTCATCATTGA dTdT-3′; reverse, 3′-dTdT CATTCGACCAGTAGTAACT-5′.

### qRT-PCR assay

The cells were incubated and starved without FBS for 24 h and then cultured in medium that was not supplemented or supplemented with arecoline for 48 h. Total RNA was extracted using TRIzol reagent (TaKaRa Bio, Shiga, Japan). The PrimeScript™ RT reagent Kit (TaKaRa Bio) was used to perform reverse transcription. TB Green detection (TaKaRa Bio) was used to perform real-time PCR. Gkyceraldehyde-3-phosphate dehydrogenase (GAPDH) was used as a standardized control. The primers used were: TPM1, forward: 5′-GCCGACGTAGCTTCTCTGAAC-3′, reverse: 5′-TTTGGGCTCGACTCTCAATGA-3′, and GAPDH; forward: 5′-ATTC CATGGCACCGTCAAGGCTGA-3′, reverse: 5'TTCTCCATGGTGGTGAAGACGCCA-3′.

### Western blotting assay

The cells were starved in an FBS-free medium for 24 h and then cultured in the absence or presence of arecoline for 48 h. The proteins were harvested using lysis buffer containing 1% phenylmethylsulfonyl fluoride (Beyotime Bio, Wuhan, China). Protein concentrations were examined and proteins were used for sodium dodecyl sulphate-polyacrylamide gel electrophoresis. The resolved proteins were transferred to polyvinylidene fluoride membranes. The membranes were incubated with the antibodies to TPM1 (RabMAb, 1:2000, Catalog Number ab133292; Abcam, Cambridge, MA, USA), Smad2/3 (1:750, Catalog Number WL0152; Wanleibio, Shenyang, China), and p-Smad2/3 (1:500, Catalog Number WL02305; Wanleibio). Alpha-tubulin antibody (1:5000, Catalog NumberRM2007; Rayantibody, Beijing, China) was used as an internal control. Chemiluminescent signals were visualised using ECL reagents (Cwbiotech, Beijing, China).

### Cell migration assay

Transwell chambers (3422; Corning, New York, NY, USA) were incubated with DMEM medium for 1 h. Suspension of 5 × 10^5^ cells/mL from each group (200 μL) were seeded in the upper chambers. After incubation for 12 h, the lower side of each chamber were fixed in 4% formaldehyde and then stained with 0.1% crystal violet. The migration cells were imaged in five different fields per chamber using a light microscope (Olympus Corp., Tokyo, Japan).

### CCK8 assay

HaCaT cells were seeded (5 × 10^3^/well) in 96-well plates in the presence of 0.16 mM arecoline for 24, 48, and 72 h. Then, 90 μL of DMEM and 10 μL of CCK-8 reagent (Genview, Houston, TX, USA) were utilised to incubate cells for 3 h. The absorbance at 450 nm was measured using a microplate reader (Bio-Rad, Hercules, CA, USA).

### Cell cycle assay

The cells were starved without FBS for 24 h and then cultured in medium that was not supplemented or supplemented with arecoline for 48 h. The cells were collected, fixed with 70% ethanol, and stained with propidium iodide (PI). The sample cells were analysed using a FACS Calibur system (BD Biosciences, San Jose, CA, USA).

### Apoptosis

The cells were cultured and either untreated or treated with arecoline for 48 h. The cells were collected and resuspended in 1× Binding Buffer containing Annexin V-FITC and PI following the manufacturer’ s instructions. The sample cells were detected in 1 h using a FACS flow cytometer (BD Biosciences).

### Wound-healing assay

HaCaT cells were cultured and either untreated or treated with arecoline. When the cells reached 80% confluency, wound fields were created across the cell monolayer using a plastic pipette tip. The cells were incubated and five non-overlapping fields of the images were taken at 48 h.

### Statistical analysis

All data are presented as mean ± standard error of the mean (S.E.M.) using SPSS 18.0. Student’s *t*-test was used to calculate differences between groups. A *p*-value < 0.05 indicated a significant difference.

## Results

### Arecoline increases expression of TPM1

**Figure 1.**
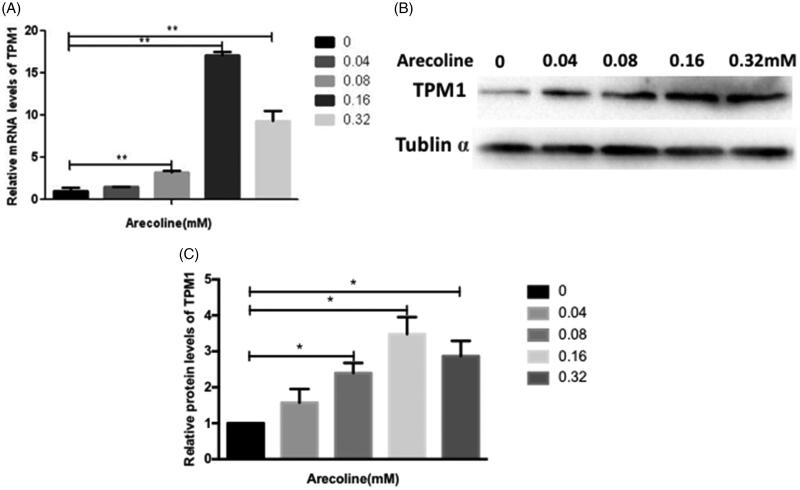
Arecoline increases the expression of TPM1. HaCaT cells were treated with increasing concentrations of arecoline for 48 h. (A) Relative TPM1 mRNA expression was determined using qRT-PCR. (B-C) TPM1 protein expression was examined using western blotting. **p < 0.01, *p < 0.05.

In our previous study, Gu et al. (2019) reported that the IC_50_ of arecoline was approximately 50 μg/mL (0.21 mM). We compared the expression of genes in untreated and arecoline-treated cells using RNA-seq. The analysis revealed a 1.5-fold increase in TPM1 in arecoline treated cells. Western blotting and qRT-PCR were used to verify the changes in TPM1 expression. The results showed that TPM1 expression was upregulated with increased concentrations of arecoline and was markedly upregulated at 0.16 mM (*p* < 0.01 and *p* < 0.05; Figure 1(A–C)). Therefore, 0.16 mM arecoline was selected for subsequent experiments.

### Arecoline suppresses cell growth and induces apoptosis

**Figure 2.**
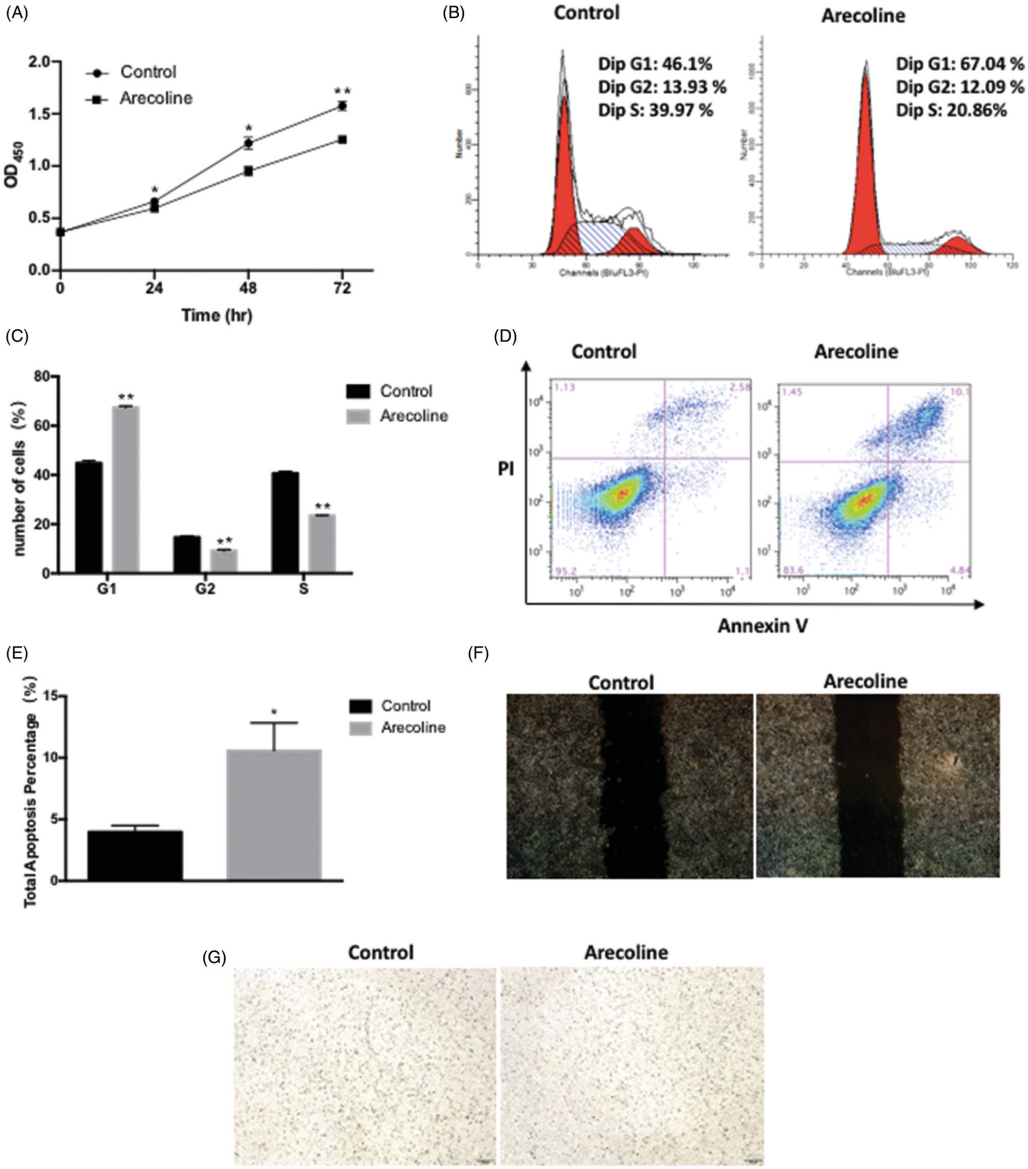
Arecoline suppresses cell growth and induced apoptosis. (A) Cells were cultured in the presence (0.16 mM) or absence of arecoline for 24, 48, 72 h. Cell proliferation was evaluated using the CCK8 assay. (B-C) Cells were cultured in the presence (0.16 mM) or absence of arecoline for 48 h, and then examined using flow cytometry. (D-E) Apoptosis was evaluated by Annexin V assay. (F) Cell migration was evaluated by a wound healing assay. (G) Cell migration was evaluated with the Transwell migration assay. The migration cells were analysed 12 h after seeding the Transwell units. **p < 0.01, *p < 0.05.

To understand the latent role of arecoline in HaCaT cells, the effect of arecoline on cell proliferation was examined using the CCK8 assay. The proliferation of cells treated with arecoline was reduced at 24, 48, and 72 h in comparison with the control (*p* < 0.01 and *p* < 0.05; Figure 2(A)). The effects of arecoline on the HaCaT cell cycle and cell apoptosis were observed using flow cytometry. After treatment of HaCaT cells with arecoline for 48 h, an increased percentage of cells in the G1 phase was observed, while fewer cells accumulated in the S phase (*p* < 0.01; Figure 2(B,C)). In addition, it was confirmed that arecoline promoted apoptosis (*p* < 0.05; Figure 2(D,E)). In addition, Transwell migration and wound-healing assays were utilized to explore the role of arecoline in HaCaT cell migration. No significant changes were observed in wound-healing assays and no cells passed through the membrane pores (Figure 2(F,G)).

Taken together, these observations revealed that arecoline suppressed the cell growth, induced cell cycle arrest at the G1 phase, and promoted apoptosis, but had no effect on migration.

### Knockdown of TPM1 using siRNA in HaCaT cells

**Figure 3.**
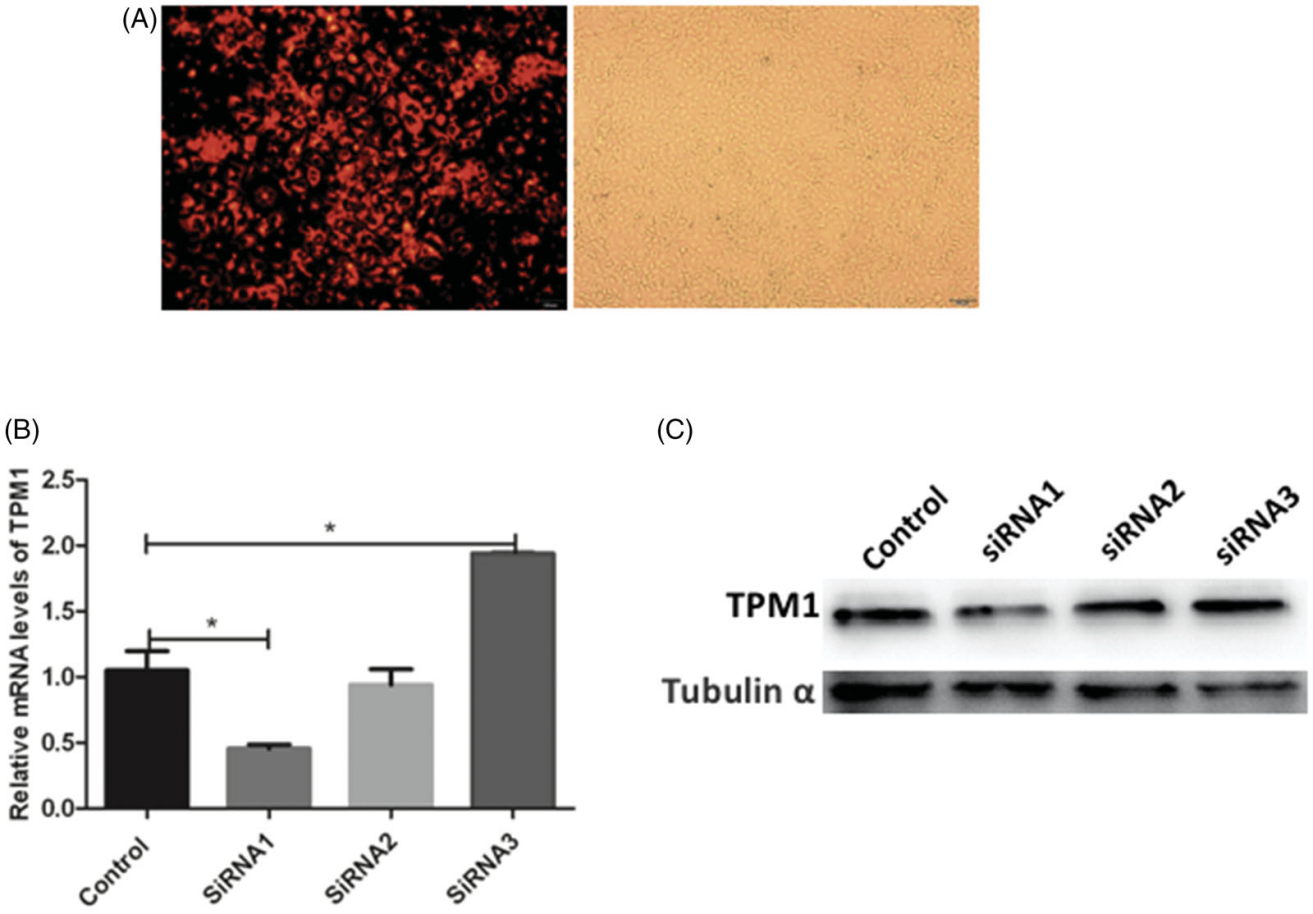
Knockdown of TPM1 using siRNA in HaCaT cells. (A) Transfection efficiency of siRNA TPM1 was tested using FAM‐small interfering RNA by fluorescence microscopy. (B) Transfection efficiency of siRNA TPM1 in HaCaT cells treated with arecoline was examined using qRT-PCR 48 h after transfection. (C) TPM1 protein expression was evaluated using western blotting 48 h after transfection. *p < 0.05.

To explore the potential function of TPM1, cells treated with arecoline were transfected with three different siRNAs to silence the expression of TPM1. After 48 h, the efficiency of transfection was assessed in the *Cy3*‐labeled negative control using fluorescence microscopy. Fluorescent particles within the cells indicated that siRNA was transfected successfully into HaCaT cells (Figure 3(A)). Western blotting and qRT-PCR revealed that siTPM1‐1 effectively reduced TPM1 protein and mRNA expression in transfected cells. In contrast, siTPM1-2 and siTPM1‐3 produced no reduction compared with the blank control (*p* < 0.05; Figure 3(B,C)). These data indicated that siTPM1-1 was effective in inhibiting TPM1. Thus, siTPM1-1 was selected for use in further experiments.

### siRNA-mediated knockdown of TPM1 attenuates the effect of arecoline on cell growth and apoptosis

**Figure 4.**
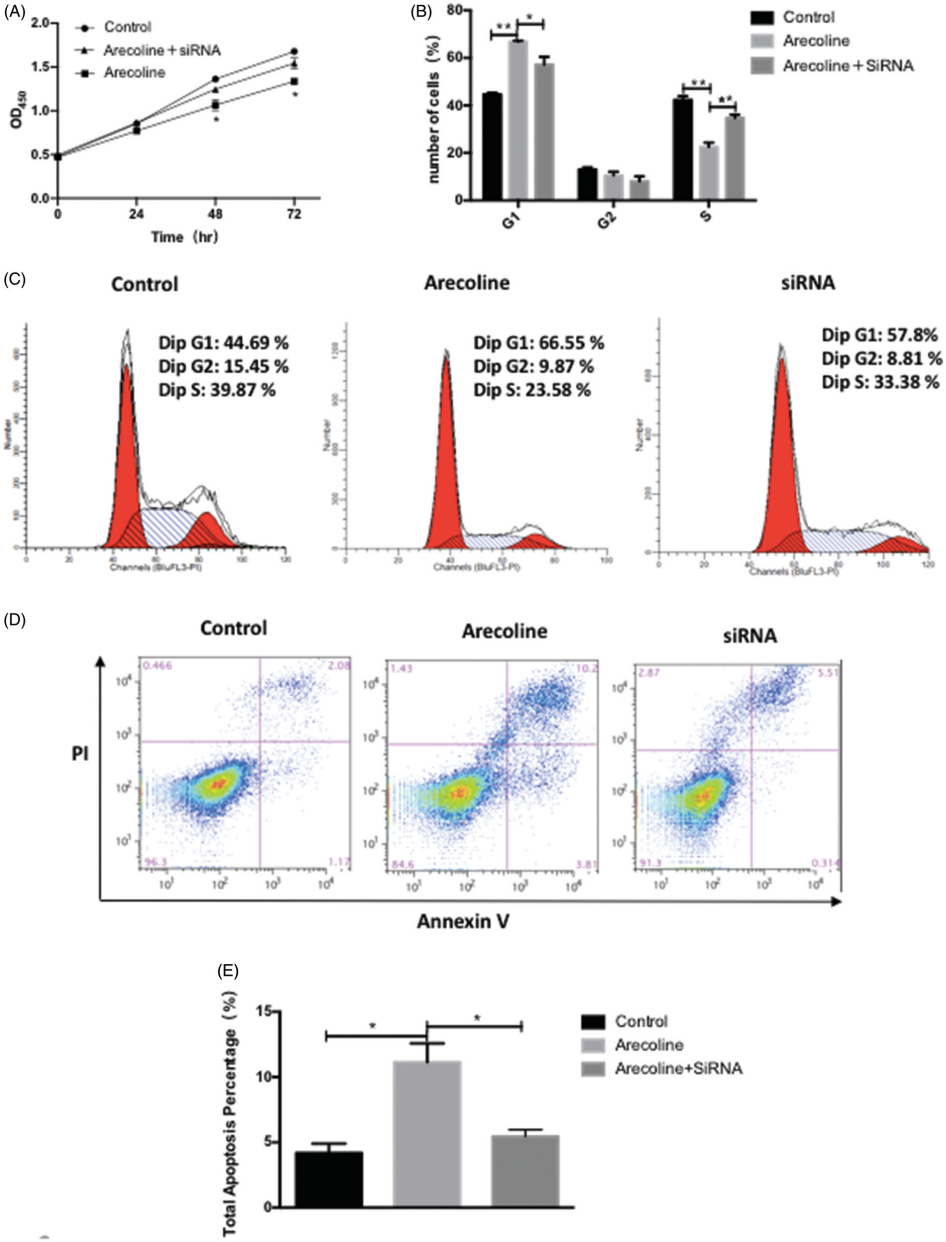
siRNA-mediated knockdown of TPM1 attenuates the effect of arecoline on cell growth and apoptosis. (A) Cells were treated with 0.16 mM arecoline for 24, 48, and 72 h. The effect of siRNA TPM1 on cell proliferation in HaCaT cells was evaluated by CCK8 assay. (B–C) Cells were treated with 0.16 mM arecoline for 48 h, and then examined by flow cytometry. (D–E) The effect of siRNA TPM1 on apoptosis of HaCaT cells was evaluated with an Annexin V assay. **p < 0.01, *p < 0.05.

To investigate whether TPM1 mediates the relevant biological behaviour of arecoline, HaCaT cells were transfected with siRNAs to silence TPM1 expression. Arecoline had less effect on reducing the proliferation of TPM1-depleted cells in the CCK8 assay (*p* < 0.05; Figure 4(A)). Flow cytometry analysis demonstrated a decreased percentage of TPM1-depleted cells in the G1 phase and an increased percentage of TPM1-depleted cells in the S phase (*p* < 0.05 and *p* < 0.01; Figure 4(B,C)). The data also confirmed that arecoline was less effective in promoting apoptosis in TPM1-depleted cells (*p* < 0.01 and *p* < 0.05; Figure 4(D,E)). Thus, knocking down TPM1 weakened the effect of arecoline on cell growth and apoptosis.

### TGF-β/Smad pathway mediates arecoline-stimulated TPM1 expression in HaCaT cells

**Figure 5.**
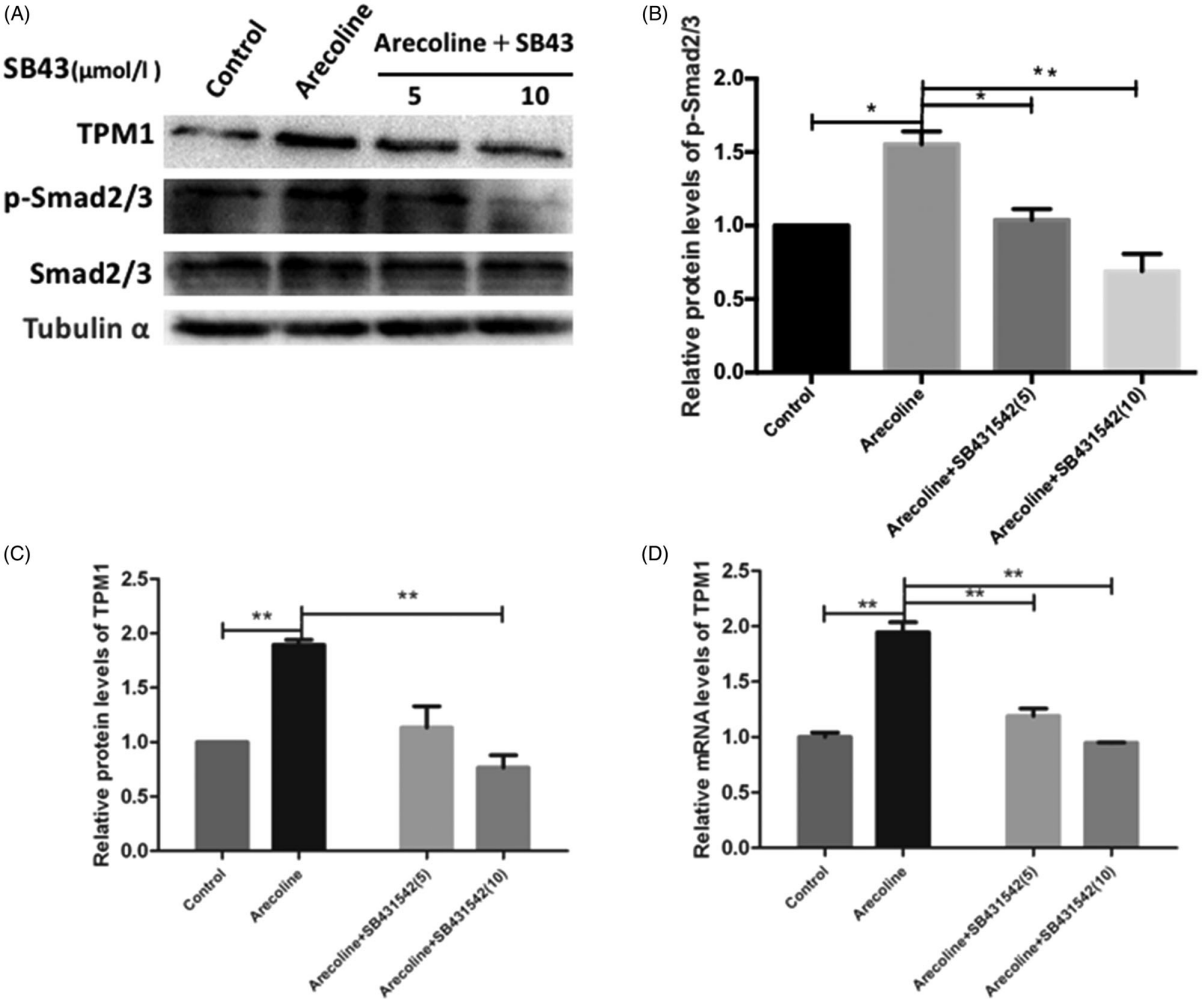
The TGF-β/Smad signalling pathway mediates arecoline-stimulated TPM1 expression in HaCaT cells. Cells were pre-treated with SB431542 at 5 and 10 μmol/L for 60 min followed by stimulation with arecoline for 48 h. (A-C) Proteins were extracted and western blotting was used to detect the expression of TPM1, p-Smad2/3, and Smad2/3. (D) mRNAs were extracted and qRT-PCR was used to examine TPM1 expression levels. **p < 0.01, *p < 0.05.

The TGF-β/Smad pathway participates in regulating TPM1 (Pawlak et al. 2004; Gervasi et al. 2012; Yang et al. 2013). We assessed whether the TGF-β/Smad pathway participated in the arecoline-mediated stimulation of TPM1. SB431542 (a selective TGF-β1 receptor inhibitor) was used to evaluate the role of the pathways in the upregulation of TPM1. After stimulation with arecoline for 48 h, the phosphorylation of Smad 2/3 in arecoline-treated cells was increased, indicating that arecoline positively regulates the TGF-β/Smad pathway. After exposure to SB431542, Western blotting and qRT-PCR demonstrated the p-Smad 2/3 and TPM1 expression were both decreased (*p* < 0.05 and *p* < 0.01; Figure 5(A–D)). These results demonstrated that the TGF-β/Smad pathway may play a crucial role in arecoline-stimulated TPM1 induction in HaCaT cells.

## Discussion

Arecoline suppressed the cell growth, caused cell cycle arrest, and induced cell apoptosis in HaCaT cells, but had no effect on migration. qRT-PCR and western blotting analyses revealed that arecoline increased TPM1 at both the transcription and translation levels. To investigate the function of TPM1 in HaCaT cells, we transfected arecoline treated HaCaT cells with TPM1 siRNA and found that the effect of arecoline on cell proliferation, apoptosis, and cell cycle arrest at the G1 phase was weakened using the CCK8 assay, cell cycle, and apoptosis analyses. Furthermore, western blotting revealed that the TGF-β/Smad pathway may play a critical role in the upregulation of TPM1 induced by arecoline.

Arecoline is considered a paramount factor in the development of OSF. In several studies, arecoline suppressed cell growth and induced an increase in the percentage of HaCaT cells remaining in the G1 phase (Gu et al. 2019; Zhou et al. 2013). Arecoline significantly suppressed HaCaT cell viability and induced cell apoptosis in another study (Li et al. 2014). Our findings are consistent with these previous reports. However, contrary to a previous study on cell migration, we observed no obvious differences between HaCaT cell lines treated with arecoline and the controls. Previous studies have shown that human oral epithelial cells treated with a low dose of arecoline for a prolonged time (90 days) displayed increased migration compared with untreated controls (Wang et al. 2016). This discrepancy may be due to the different treatment concentrations and treatment times of arecoline used in the studies.

Dysregulation of TPM1 is functionally involved in human disorders. Downregulation of TPM1 was reported to promote U2OS cell viability, migration, and invasion in osteosarcoma (Jiang et al. 2017). In renal cell carcinoma, TPM1 overexpression was found to inhibit cell proliferation and induce apoptosis in renal cancer cells (Wang et al. 2015, 2019; Tang et al. 2018). By targeting TPM1, downregulation of miR-21–5p inhibited human aortic vascular smooth muscle cell proliferation and migration (Jia et al. 2019). However, the role of TPM1 in fibrogenesis has not yet been defined. We demonstrated that TPM1 weakened the effect of arecoline on cell growth, apoptosis, and cell cycle in HaCaT cells.

TGF-β/Smad signalling is related to the induction of TPM1. TGF-β mediates the induction of TPM1 in normal and metastatic epithelial cells via TGF-β/Smad signalling (Bakin et al. 2004). TGF-β/Smad signalling upregulates JunB and is involved in the regulation of tropomyosin in kidney and mammary epithelial cells (Gervasi et al. 2012). The sequence analysis of human TPM1 promoter showed two regions with putative Smad-binding elements (SBEs) that are located upstream of TATA-box. Chromatin-immunoprecipitation revealed that TGF-β1 increases the occupation of both SBEs by p-Smad 2/3. These indicate that TGF-β stimulates binding of Smad transcription factors to the TPM1 promoter (Safina et al. 2009). Arecanut-induced TGF-β signalling in epithelial cells and activation of TGF-β signalling was speculated to be the main cause of increased collagen production in OSF (Moutasim et al. 2011; Khan et al. 2012; Wang et al. 2018). In our study, we chose SB431542 to examine the role of TGF-β/Smad signalling in the regulation of TPM1 by arecoline. TPM1 was significantly decreased through inhibition of the TGF-β/Smad pathway.

## Conclusions

Knockdown of TPM1 attenuated the suppression of arecoline on cell viability in HaCaT cells. The findings may help to better define the role and mechanism of arecoline in OSF progression. TPM1 may be a valuable molecular target for treating OSF.
